# A Pilot Study of Laparoscopic Doppler Ultrasound Probe to Map Arterial Vascular Flow within the Neurovascular Bundle during Robot-Assisted Radical Prostatectomy

**DOI:** 10.1155/2013/810715

**Published:** 2013-06-19

**Authors:** Ketan K. Badani, Edan Y. Shapiro, William T. Berg, Sarah Kaufman, Ari Bergman, Chris Wambi, Arindam RoyChoudhury, Trushar Patel

**Affiliations:** ^1^Department of Urology, Columbia University College of Physicians and Surgeons, 161 Fort Washington Avenue, 11th Floor, New York, NY 10032, USA; ^2^Department of Biostatistics, Columbia University College of Physicians and Surgeons, 722 West 168th Street, 6th Floor, New York, NY 10032, USA

## Abstract

*Purpose.* To report on the feasibility of a new Laparoscopic Doppler ultrasound (LDU) technology to aid in identifying and preserving arterial blood flow within the neurovascular bundle (NVB) during robotic prostatectomy (RARP). *Materials and Methods.* Nine patients with normal preoperative potency and scheduled for a bilateral nerve-sparing procedure were prospectively enrolled. LDU was used to measure arterial flow at 6 anatomic locations alongside the prostate, and signal intensity was evaluated by 4 independent reviewers. Measurements were made before and after NVB dissection. Modifications in nerve-sparing procedure due to LDU use were recorded. Postoperative erectile function was assessed. Fleiss Kappa statistic was used to evaluate inter-rater agreement for each of the 12 measurements. *Results.* Analysis of Doppler signal intensity showed maintenance of flow in 80% of points assessed, a decrease in 16%, and an increase in 4%. Plane of NVB dissection was altered in 5 patients (56%) on the left and in 4 patients (44%) on the right. There was good inter-rater reliability for the 4 reviewers. Use of the probe did not significantly increase operative time or result in any complications. Seven (78%) patients had recovery of erections at time of the 8-month follow-up visit. *Conclusions.* LDU is a safe, easy to use, and effective method to identify local vasculature and anatomic landmarks during RARP, and can potentially be used to achieve greater nerve preservation.

## 1. Introduction

Erectile function after radical prostatectomy is predicated upon a number of factors, including the ability to carefully dissect and separate the cavernous nerves from the prostate [1–6]. Despite offering improved magnification of the operative field and precise surgical instrumentation, improvement in potency rates after robotic-assisted radical prostatectomy (RARP) remains of great interest, and there have been several efforts to introduce new nerve-sparing techniques to improve postoperative potency [7–9]. However, the reproducibility of these results remains controversial, with subsequent reports finding no difference in potency rates regardless of the nerve-sparing technique [10]. Regardless, visualization continues to be a hindrance in preserving the nerves and vessels around the prostate, and there remains a need for more accurate methods to correctly identify and preserve the neurovascular bundle (NVB) during surgery.

There have been different attempts to provide better visualization of the NVB during dissection, including the use of intraoperative transrectal ultrasound (TRUS) to help improve visualization of the NVB during prostatectomy and provide real-time feedback in assisting precise nerve dissection in patients with suspected extracapsular extension [11, 12]. However, the TRUS technique can potentially be invasive, time consuming, and cumbersome in nature. Other technologies have focused on nerve stimulation, intracavernosal injection of fluorescent tracers, and other novel imaging techniques [13–16]. While promising, these techniques are all complicated by a long FDA approval process, limited availability of technology, learning curve, and high expense associated with their adoption. 

In contrast, laparoscopic Doppler ultrasound (LDU) technology has been utilized in a variety of urologic procedures as a widely available technology at a minimal cost and is not associated with a learning curve. The benefits of LDU to precisely identify blood vessels have previously been shown in partial nephrectomy, pyeloplasty, and laparoscopic varicocelectomy [17–21]. This technology is a potential, noninvasive alternative to TRUS that provides real-time audio feedback on the degree of arterial blood flow, which may facilitate greater precision in maintaining local vasculature and establishing the planes of dissection. Despite being widely available, time efficient, and cost effective, LDU has not previously been used robotically during RARP. In a prospective feasibility pilot study, we examined the ability of an LDU probe to provide confirmation of NVB location with the aim of preserving arterial blood flow within the NVB during RARP.

## 2. Materials and Methods

After receiving Institutional Review Board approval, a total of 9 patients were enrolled in this prospective feasibility pilot study. Prior to surgery, preoperative erectile function was assessed using the Sexual Health Inventory for Men (SHIM) questionnaire. Only those with baseline normal potency (SHIM composite score ≥21) were eligible for the study. Additional inclusion criteria were a plan for bilateral nerve sparing and low to intermediate risk disease by the D'Amico classification. Patients were excluded if they had diabetes or peripheral vascular disease. All patients underwent RARP by a single surgeon (KKB). Patient demographic, intraoperative, and clinicopathologic data were prospectively collected.

**Figure 1:**
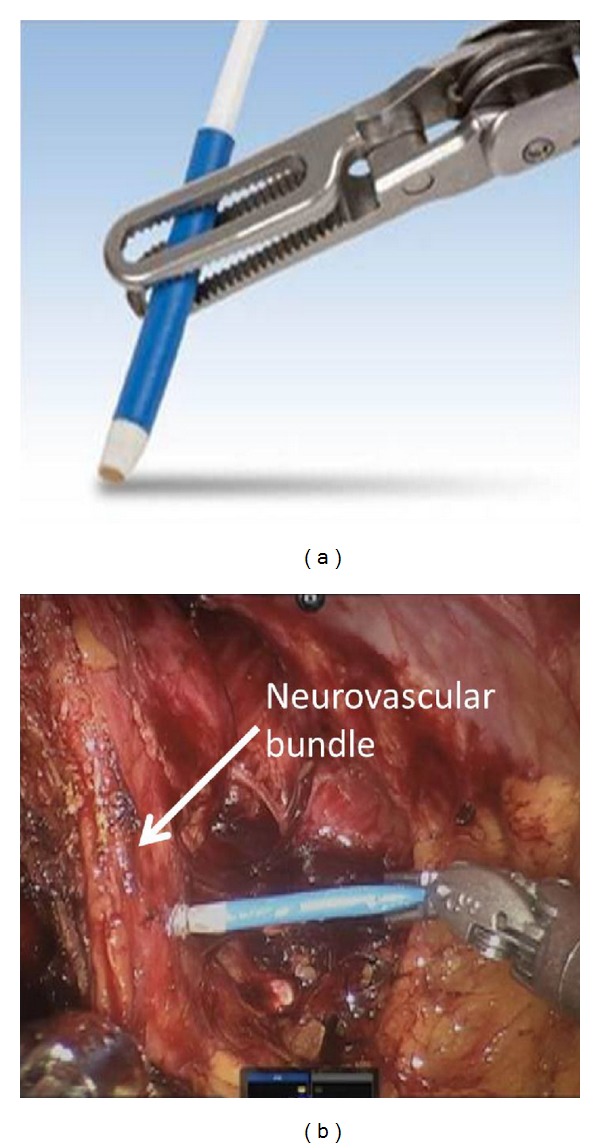
Demonstration of probe manipulation with robotic arm (a). Example of intraoperative manipulation (b).

**Figure 2:**
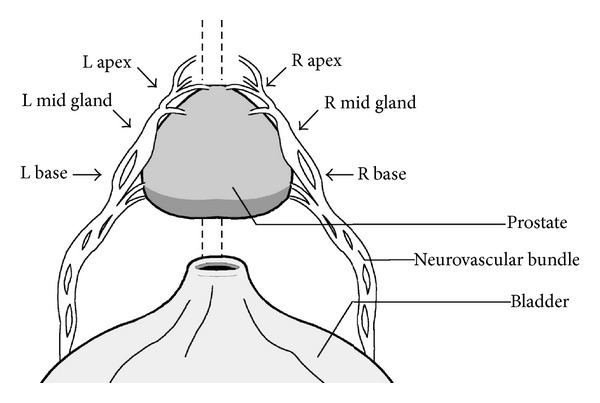
Diagram of neurovascular bundle and points assessed with LDU probe.

The RARP was performed as previously described [22]. Prior to performing the nerve-sparing dissection of the procedure, a 20 mHz laparoscopic single-use ultrasound Doppler probe (Vascular Technology Inc., Nashua, NH, USA) was introduced into the pelvis through the 12 mm assistant port (Figure 1). The probe was then manipulated by the console surgeon using a robotic needle driver. The surgeon carefully placed the probe upon six prespecified locations of the NVB—the base, midgland, and apex of the prostate, bilaterally, in order to trace its course along posterolateral aspects of the prostate (Figure 2). Since this was a pilot study, these 6 locations were selected with the aim of representing discrete areas along the expected course of the NVB from base to apex and standardizing the procedure for the purpose of this study. The probe was attached to an amplified speaker system that was audible throughout the operating room. The degree of Doppler flow at each point was subjectively evaluated by the console surgeon and 3 independent assessors. Based on the intensity of the Doppler auditory signal, each point was described as “absent,” “low,” “moderate,” or “high.” Following the initial six measurements, the probe was removed from the pelvis, and the neurovascular bundle was carefully dissected from the prostate. Nerve dissection was performed using either the standard or lateral prostatic fascia-sparing technique. This decision was based on cancer location from the preoperative 12-core TRUS biopsy [23]. During the nerve-sparing dissection, the console surgeon documented the type of nerve sparing technique (standard versus lateral) completed and whether this was modified from the preoperative plan following the intraoperative Doppler mapping at the 6 measured locations. In addition, the surgeon documented whether the limits of dissection were altered following the Doppler findings. After the prostate was removed from the pelvis, the LDU probe was reintroduced and the same 6 anatomic locations were reexamined. Each assessor then rated signal strength again, based on the same scale. Patient arterial pressure was monitored intra-operatively by brachial artery cuff and maintained above 100/50 during LDU use. Correlation between pre- and postdissection LDU intensity scores was used to determine if there was a decrease, maintenance, or increase in signal strength after NVB dissection. Primary outcome measures were feasibility and ease of use of the LDU probe. For the purpose of the study, feasibility was defined as the ability to easily record and trace arterial flow within the NVB, while ease of use relates to whether use of LDU probe significantly extended the length of the procedure and/or resulted in any intraoperative complications. Secondary outcome measures included modification of the surgical plan, with respect to nerve-sparing technique, maintenance of arterial blood flow after dissection, and postoperative potency rates, as measured by erections suitable for intercourse.

Categorical variables were assessed using *χ*² test, with continuous nonparametric data analyzed through the paired Wilcoxon test. The Fleiss interrater reliability analysis using the Kappa statistic was performed to determine consistency among raters. A *P* value of less than 0.05 was considered to be significant. All statistical analysis was done using STATA 11.0 (Stata Corp, TX, USA).

## 3. Results

 Baseline demographic data can be found in Table 1. Mean age was 57.6 years (53.4–69.9). Mean preoperative PSA value was 4.1 (1.2–8.0). All nine men were clinical stage T1c. According to D'Amico risk classification, three men were low risk and six were intermediate risk. All patients had normal erectile function at baseline, with a mean SHIM score of 23.7 (range 21–25).

 Operative information is listed in Table 2. There was no significant difference in mean flow intensity in pre- versus postdissection at 5 of the 6 locations (Table 3). Further analysis of Doppler signal intensity showed maintenance in arterial flow intensity at 80% of points assessed with a decrease in only 16% of points. Interestingly, 4% of the points demonstrated an increase in flow following NVB dissection. Overall, the LDU was found to be safe and only added a mean of 8.2 minutes to the entire procedural length (*P* = NS). 

A lateral prostatic- (interfascial-) sparing dissection was performed on the right side in 5 patients and on the left in 2 patients (Table 2). While using the LDU probe, the limits of the dissection were altered in 44% and 56% of patients on the right and left sides, respectively. These alterations to the lateral height of dissection were based on previously nonvisualized arterial vessels that were only recognized intraoperatively through use of the LDU and then accommodated into the anterior limits of the dissection plane. 

The Fleiss interrater reliability Kappa statistic found good agreement between all raters in 9 of the 12 locations measured (Table 4). The raters did not agree for the following locations: right midgland before dissection (Kappa = 0.439, *P* < 0.001), left base after dissection (Kappa = 0.351, *P* = 0.003), and left apex after dissection (Kappa = 0.212, *P* = 0.04). Although functional outcomes were not a primary endpoint of the study, at the 8-month postoperative time point, 7 patients (78%) had recovery of erections that were subjectively “suitable for sexual intercourse”; notably, 4 of these men had erections rated as back to baseline function. Of note, all men were immediately placed on postoperative penile rehabilitation involving phosphodiesterase-5 inhibitors and vacuum erection devices. 

## 4. Discussion

Use of an LDU probe during nerve sparing in RARP is a novel way to supplement existing nerve-sparing techniques. Despite the higher magnification, which makes locating anatomic landmarks easier with RARP, visual perception alone cannot provide critical information about unique underlying vasculature [7]. This lack of anatomic information allows for the possibility of inadvertent nerve damage during prostatectomy. LDU use provides information with regard to the relative levels of arterial blood flow in the NVB, allowing the surgeon to better demarcate the nerve tract, enhance precision when establishing a plane of dissection, and minimize unnecessary damage of tissue. Our use of the LDU device in this pilot study facilitated high levels of maintenance of arterial blood flow in the NVB and potentially obviated vasculogenic erectile dysfunction.

Prior attempts to use ultrasound technology (i.e., TRUS) during laparoscopic radical prostatectomy to gain real-time visualization have proved to be cumbersome and difficult, either requiring a human assistant to manipulate the probe while maintaining intraoperative sterility or a specialized robotic probe holder [11, 12, 24]. A unique advantage of the LDU probe is that it is small enough to pass through the 12 mm assistant port during RARP. Additionally, in the robotic system, the LDU probe is easily manipulated by the console surgeon without the need for bedside assistance. Moreover, there does not appear to be a learning curve associated with LDU used based on its implementation without a significant increase in the length of surgery. 

 Ultimately, the clinical applicability of using LDU to perform a better nerve-sparing technique is to preserve the nerves responsible for erectile function. It is well known that both preoperative and intraoperative factors have been significantly associated with affecting postoperative return to potency. Patient factors such as age, prostate volume, preoperative erectile dysfunction, low serum testosterone level, or history of diabetes mellitus are examples reported in the literature that significantly affect potency outcomes after radical prostatectomy [25–28]. Despite preoperative knowledge of patient's demographics and clinical characteristics, it remains difficult to predict the degree of success and the timeframe required to recover erectile function. Furthermore, several intraoperative factors impact return of erectile function following radical prostatectomy. This includes minimal traction, diminished use of electrocautery, and identification of correct dissection planes [28]. Though both clinical and operative factors affect postoperative potency outcomes, we believe that the intraoperative handling of the NVB plays a far more significant role in how men recover after RARP. For these patients, minimizing irrecoverable damage to the NVB is essential during their nerve-sparing procedure [29]. 

A recent study reported that the sheer attempt alone to spare the nerves is significantly predictive of higher potency outcomes [28]. Bradford et al. have placed importance on minimizing traction and handling of tissue over any specific nerve-sparing technique employed, while multiple recent studies have also reported that a surgeon's own perception of nerve-sparing quality can be predictive of better potency outcomes [26, 27]. These studies highlight the subjectivity that arises from a surgeon's own interpretation of a successful nerve-sparing procedure, as well as the need for more objective measures. 

Our pilot feasibility study confirms the ease of using LDU to facilitate the nerve-sparing procedure in RARP. Intraoperative LDU use during RARP was safe and feasible in all patients. This is likely due to the small size of the probe, allowing it to be easily inserted through a robotic assistant port and carefully manipulated with the robotic instruments. Even though the TRUS technique has the theoretic advantage of being able to visualize the apex without being hindered by the tight space within the pelvis, we did not encounter technical difficulty using the LDU probe at the apex, likely due to the combination of the small flexible probe and the endowrist manipulation and enhanced visualization inherent to the robotic platform. Additionally, the use of A-mode Doppler measurements, projected via a handheld speaker, allowed for subjective quantification of flow by all members of the operating team. Although there was good internal reliability of the measure at 9 of the 12 locations, suggesting that the LDU appears to be uniformly measured by multiple judges, the lack of quantitative data (e.g., B-mode Doppler flow) or ability to measure a waveform, as can be done with TRUS Doppler, remains a clear limitation that must be considered. Despite this drawback, in our initial experience LDU aided in locating areas to be preserved, and its easy manipulation by the console surgeon helped give attention to more delicate areas, while minimizing any unnecessary traction or handling. Additionally we found that its use allowed the surgeon to alter the nerve dissection based on arterial flow patterns. 

Although this was only a pilot feasibility study, and we demonstrated safety and feasibility of a novel application, other limitations should be mentioned. First, this represents a single surgeon's experience at a single institution with a small volume of patients. However, due to the ease of LDU use with the robotic system with no added learning curve, it is likely that its use will provide substantial benefit for all surgeons, irrespective of specific nerve-sparing technique employed or prior experience. 

Another limitation of this study is the sole use of A-mode ultrasound. A recent study drawn upon the initial description by Ukimura et al. used the B-mode with TRUS to create a 3D reconstruction of the prostate and its neurovascular bundle [11, 30]. While this method was shown to be safe and feasible within the confines of their cohort, it required expensive additional hardware and software to use. Moreover, they note that actual visualization of the NVB is often not possible and therefore rely on the preservation of the blood vessels in the posterolateral region as a surrogate for the preservation of the cavernous nerves. In contrast, our use of the LDU provides similar information in a more cost-effective manner (reusable Doppler transceiver is $998; single-use probes are $195/each), with a shorter learning curve and with a less cumbersome and invasive setup. 

A third potential limitation of our LDU use is lack of visualization of potential cancerous lesions within the prostate. A recent study by Hung et al. utilized TRUS to identify hypoechoic lesions with the prostate to improve their oncologic control during dissection [31]. In their study of 10 patients, they were able to obtain 90% negative margins through use of the TRUS. Interestingly, the 10% positive margin rate is similar to the rates reported in the literature without the use of the TRUS system. Additionally, only half of the patients even had visible lesions on TRUS that were potentially cancerous, and none had tissue confirmation of these lesions. As previously stated, our feasibility trial using the LDU probe was primarily an effort to improve identification and preservation of the neurovascular bundle, and decisions regarding oncologic control should always take precedence over decisions to spare the NVB, although excellent oncologic results and negative margins have been achieved using the robotic system alone. Although the LDU system is not intended to improve oncologic control, we believe that this technology is a potential tool to enhance the ability of the operating surgeon to achieve enhanced bilateral nerve sparing.

Another limitation is the potential difficulty in measuring flow at the apex using the LDU probe. As mentioned previously, this did not appear to be technically challenging; however, of the 3 anatomic locations measured, flow at the apex was reduced bilaterally, even before dissection. Since apical flow may serve as a prognostic indicator of postoperative erectile function, it is important to ensure that arterial flow can be accurately measured at this location. Perhaps with more experience, this difference will be mitigated. Thus, a larger cohort of patients in a randomized controlled trial model, with long-term potency followup, are needed to better assess this modality. It is also difficult to objectively quantify how the boundaries of dissection were altered based on the results of the LDU use. This limitation further limits any definitive conclusions from this study. 

Lastly, there is a limitation regarding the lack of long-term potency outcomes. Regardless of whether the surgeon subjectively appreciated the anatomic location of the NVB to a greater extent or altered the nerve-sparing plane based on LDU, it is unclear if this results in differences in functional outcomes. Although not all patients completed the SHIM questionnaire postoperatively, of the 9 patients in the study, 78% had subjective return of erection within an 8-month time point. It is possible that with further followup, patients without return of baseline erectile function will, in fact, return to their baseline status. Overall, the promising results of this feasibility study warrant further followup to evaluate functional endpoints of potency outcomes. 

## 5. Conclusions

Our pilot study confirms the safety, ease of use, and feasibility of using LDU during RARP. This novel technique provides information for identifying and maintaining local vasculature in the NVB thereby potentially facilitating improved potency recovery after radical prostatectomy. These early results are encouraging, and future studies with a larger cohort and long-term potency data are needed to fully assess the impact of LDU on outcomes.

## Figures and Tables

**Table 1:** Baseline demographic and clinicopathologic characteristics.

Characteristic	Population
Patients (*n*)	9
Mean age (range)	57.6 (49–70)
Preoperation SHIM (range)	23.7 (21–25)
Clinical stage	
cT1c	9
Biopsy Gleason score (%)	
≤6	3
7	6
≥8	0
Preoperation PSA (range)	4.1 (1.2–8.0)
D'Amico risk group	
Low	3
Intermediate	6
High	0

**Table 2:** Intraoperative data.

Operative time (min)	156 (122–185)
EBL (mL)	57.6 (49–70)
Time for probe (min)	8.2 (5–13)
Right NVB dissection (*n*)	
Standard	4
Lateral prostatic fascia sparing [23]	5
Right NVB dissection (*n*)	
Standard	7
Lateral prostatic fascia sparing [23]	2
Change in dissection based on LDU (*n*)	
Left	5 (55%)
Right	4 (44%)

**Table 3:** Mean arterial flow intensity using LDU probe.

Point of measurement	Pre (*μ*)	Post (*μ*)	*P* value
Right base	2.19	2.61	0.11
Right midgland	2.04*	1.84	0.93
Right apex	2.10	1.97	0.61
Left base	2.14	1.83*	0.05
Left midgland	1.78	1.73	0.80
Left apex	1.80	1.68*	0.83

*There was no interrater agreement at these locations on the Fleiss Kappa statistic.

**Table 4:** Interrater reliability.

Point of measurement	Kappa	*P* value
Predissection		
Right base	0.160	0.232
Right midgland	0.168	0.195
Right apex	0.439	<0.001*
Left base	0.122	0.324
Left midgland	0.124	0.227
Left apex	0.116	0.292
Postdissection		
Right base	0.086	0.523
Right midgland	0.351	0.003*
Right apex	0.172	0.142
Left base	0.191	0.108
Left midgland	0.188	0.060
Left apex	0.212	0.042*

*There was no interrater agreement at these 3 locations.
